# PsychStart: a novel mentoring scheme for supporting and valuing medical students interested in psychiatry

**DOI:** 10.1192/bjb.2020.107

**Published:** 2021-12

**Authors:** Thomas Hewson, Nikki Thomas, Kate Lovett, Helen Bruce, Derek K. Tracy

**Affiliations:** 1 Pennine Care NHS Foundation Trust, UK; 2 Cambridgeshire and Peterborough NHS Foundation Trust, UK; 3 Nottinghamshire Healthcare NHS Foundation Trust, UK; 4 Royal College of Psychiatrists, London, UK; 5 Livewell Southwest, Plymouth, UK; 6 East London NHS Foundation Trust, UK; 7 Great Ormond Street Institute of Child Health, University College London, UK; 8 Oxleas NHS Foundation Trust, London, UK; 9 Department of Psychosis Studies, Institute of Psychiatry, Psychology and Neuroscience, King's College London, UK

**Keywords:** Mentoring, medical education, education and training, recruitment, medical student

## Abstract

We describe the establishment and evaluation of a career-based mentoring scheme (PsychStart) for medical students interested in psychiatry. Medical students reported multiple benefits of mentoring, including enhanced personal and professional development, increased career and clinical knowledge, and broadened exposure to psychiatry. The mentoring scheme was also found to promote and sustain interest in the specialty. Further evaluation is required to determine the long-term effects of mentoring and how this may compare with other undergraduate enrichment activities. We conclude that mentoring in psychiatry could offer innovative solutions for improving recruitment and retention, and for supporting and valuing medical students who demonstrate an early interest in the specialty.

Increasing the number of doctors choosing to enter and remain in psychiatry has been a key health policy priority in the UK over the past 3 years.^1,2^

The proportion of graduates from each medical school entering immediately into psychiatry post-foundation training varies widely, from 0.1 to 0.4%.^3^ Overall, approximately 5% of foundation year 2 (F2) doctors who enter directly into specialty training are appointed to core psychiatry training each year.^3,4^ However, increasingly large numbers of junior doctors are delaying their progression into specialty training, for multiple complex reasons.^3,4^ Although recruitment to core training has improved more recently, almost 10% of core and consultant posts still remained unfilled in 2019.^5,6^

Recruiting and retaining sufficient doctors to fill these gaps requires multifaceted, long-term approaches. As well as increasing the number of doctors entering medical school, policy has recently focused on improving the exposure and experience of psychiatry training at undergraduate level.^7^ Prior research has demonstrated that regular early undergraduate exposure to psychiatry (through the Psychiatry Early Experience Programme, PEEP) can sustain positive attitudes towards psychiatry and challenge preconceptions about the specialty.^8^ However, little is known about the value of mentorship for medical students interested in psychiatry, despite this demonstrating clear value for students and doctors in other specialties and the broader literature.^9–12^

This paper describes and evaluates the establishment of an undergraduate enrichment programme that combines opportunities for mentorship with additional clinical exposure to psychiatry. To our knowledge, this is the first paper to explore the potential role of undergraduate mentorship in improving recruitment to the profession. We also evaluate the potential utility of mentoring for supporting, and promoting the development of, the future psychiatric workforce.

## Method

PsychStart, a career-based mentoring scheme for medical students interested in psychiatry, was co-founded by two of the authors (T.H. and N.T.) at the University of Nottingham in January 2018.

Medical students from all year groups were recruited to the scheme via communication through the student-led psychiatry society Mind Matters, social media advertising (Facebook and Twitter) and signposting during lectures and psychiatry teaching.

There are approximately 280 students in undergraduate years 1 and 2 of the Nottingham medical course, and 100 students in graduate-entry years 1 and 2. The undergraduate and graduate students merge during year 3, with approximately 380 students in years 3–5 of the medical programme. The psychiatry module is taught in year 4, and approximately 45 students complete this module at any one time.

Mentors were recruited from three local healthcare trusts that provide clinical psychiatry placements, as well as from the University of Nottingham and the university's Institute of Mental Health, facilitating the involvement of both academic and clinical psychiatrists. Mentor recruitment was limited to registrars (specialty trainee year 4 and above, ST4+) and consultant psychiatrists across a wide range of psychiatric subspecialties.

Places on the scheme were allocated on a first-come-first-served basis when appropriate mentors became available. Medical students were matched 1:1 or 2:1 to registrar or consultant psychiatrist mentors according to three main criteria: subspecialty interests, geographical location of mentors’ workplaces and students’ placements locations, and other career interests (e.g. in research, management or education).

All mentors and mentees were invited to attend a 2 h training session, with scheduled time incorporated for the initial ‘meet and greet’ between mentors and mentees. The mentor training was led by a consultant psychiatrist with experience of medical education and the Nottingham psychiatry module (N.T.), with input from the Director of Student Wellbeing. Mentor training topics covered the role of a mentor, theories of mentoring, characteristics of positive and negative mentorship, the structure and scope of the PsychStart scheme, the context of psychiatry at Nottingham medical school, and student welfare and support. Mentee training was coordinated by a junior doctor (T.H.) and/or the President of the student-led PsychStart committee, with oversight from a consultant psychiatrist (N.T.). Mentee training topics included the role of a mentee, characteristics of positive and negative mentoring interactions, the structure and scope of the PsychStart scheme, and career pathways and opportunities in psychiatry.

No minimum or maximum restrictions were placed on the frequency of mentor–mentee contact, but monthly email contact was recommended initially to promote engagement.

A generic email account was created for general communication and administrative queries. A separate email account monitored by a consultant psychiatrist (N.T.) was used for communication of any mentoring difficulties or issues requiring senior input.

Regular surveys were distributed to mentees to evaluate the different mentoring relationships and identify any requiring extra support. Any failing or inactive mentoring relationships, as identified by survey data and communication with the scheme's co-founders, were discussed with the concerned mentors and mentees. Where appropriate, some mentoring relationships were subject to reallocation.

To celebrate successes on the scheme and support the network, annual awards evenings were established where mentoring achievements were formally recognised. Awards were selected using information from regular feedback surveys, and mentors and mentees were also given the opportunity to submit nominations.

The data presented in this paper are from the 12- and 18-month evaluation surveys in January and June 2019 (Appendix 1 in the supplementary material, available at https://doi.org/10.1192/bjb.2020.107) and other feedback, including nominations for the annual scheme awards. All participants gave informed consent for their anonymised data to be included in future research and promotional materials. As this was a service evaluation to help improve a novel mentoring scheme, ethical approval was not required.

## Results

In mid-2020, there are currently 66 active mentoring relationships participating in the PsychStart scheme at Nottingham University. Of these, 36 are 1:1; the remaining 30 students are matched 2:1 to mentors. There are also 20 medical students on the reserve list awaiting mentor allocation.

### Mentee demographics

Of the current mentees, in mid-2020, 50 (75.8%) are women and 16 (24.2%) are men. Just under half (29; 43.9%) are in the pre-clinical phase of the medical course, with the rest in years 3 and above (37; 56.1%) (Table 1). The scheme is most popular among fourth year students (14; 21.2%) (Table 1).

**Table 1:** Year groups of medical students on the PsychStart scheme across the East Midlands in mid-2020

Year group	PsychStart mentees, *n* (%)
Undergraduate year 1	5 (7.6%)
Graduate-entry year 1	9 (13.6%)
Undergraduate year 2	8 (12.1%)
Graduate-entry year 2	7 (10.6%)
Year 3^a^	12 (18.2%)
Year 4	14 (21.2%)
Year 5	10 (15.2)
Foundation years	1 (1.5%)

a. The graduate-entry medicine (GEM) students merge with the undergraduate students from year 3 onwards.

### Psychiatric subspecialties

**Fig. 1:**
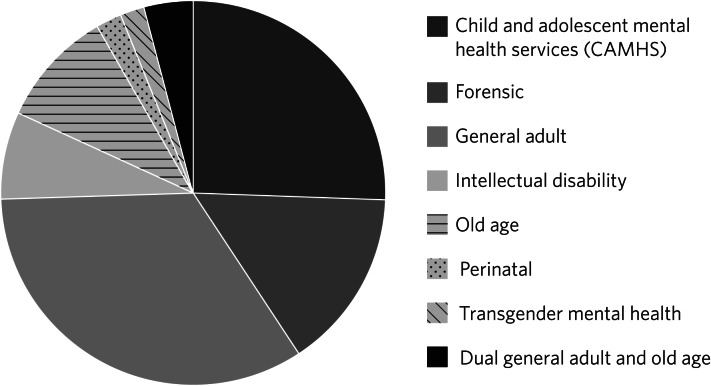
Specialty distribution of mentors on the PsychStart scheme across the East Midlands in mid-2020.

In mid-2020, there are 51 registrar and consultant psychiatrists participating in the PsychStart scheme across the East Midlands. The mentors span a range of specialties, from forensic to perinatal psychiatry, with most mentors in general adult and child and adolescent mental health services (Fig. 1).

### Survey responses

We received responses from 31 out of 44 mentees (70.5%) in the 18-month survey and 47 out of 68 (69.1%) in the 1-year survey, creating a cumulative total of 78 responses from 68 mentees; 18 students completed both surveys.

Most students had participated in the PsychStart scheme for over a year at the time of survey completion (46; 59.0%); 14 students (17.9%) had participated in the scheme for 6–12 months; and 18 (23.1%) for less than 6 months.

### Subjective ratings of scheme experiences and mentoring relationships

Including all 78 survey responses, over 90% of students described their overall scheme experience as ‘good’ or ‘excellent’ (Table 2). The organisation of, and support available on, the PsychStart scheme were also rated as ‘good’ or ‘excellent’ by 93.6 and 89.8% of mentees respectively, with no student rating these as ‘poor’ or ‘very poor’ (Table 2).

**Table 2:** Subjective mentee ratings of their experiences on the PsychStart scheme

Rating category	Proportion of mentees selecting each rating, %				
Excellent	Good	Average	Poor	Very poor
Overall PsychStart experience	38.5	52.6	7.7	1.3	0
Organisation of PsychStart	56.4	37.2	6.4	0	0
Support available through PsychStart	46.2	43.6	10.3	0	0
Quality of individual mentoring relationship	34.6	47.4	12.8	6.1	0

Over 80% of students subjectively rated the quality of their mentoring relationships as ‘good’ or ‘excellent’ (Table 2). Only 6.1% of students reported poor mentoring relationships (Table 2).

Over 80% of mentees ‘agreed’ (48.2%) or ‘strongly agreed’ (35.7%) that they had been well-matched to their mentors; 14.3% of responses were neutral and 1.8% disagreed.

### Frequency of mentee–mentor contact

**Fig. 2:**
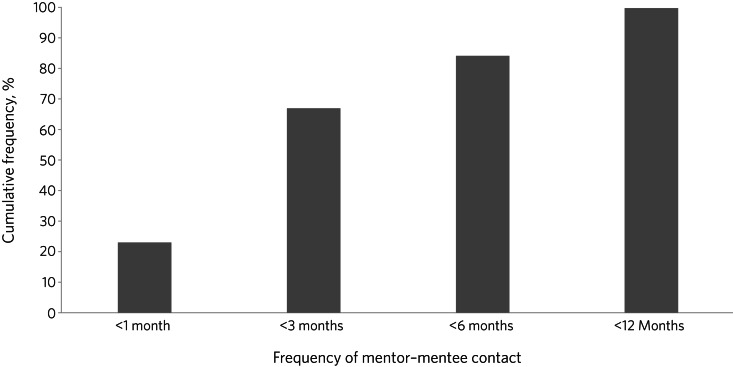
Frequency of mentor contact reported by mentees on the PsychStart scheme.

Two-thirds of survey responses indicated a frequency of mentee–mentor contact of every 3 months or more often (52; 66.7%), of which just under a quarter (18; 23.1%) reported monthly contact with mentors (Fig. 2). Over 80% (66; 84.6%) were in contact with their mentors every 6 months or more often (Fig. 2).

### Mentoring activities

**Fig. 3:**
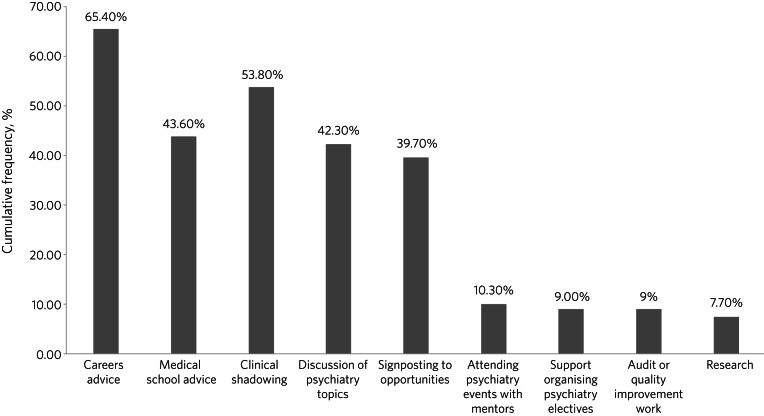
Frequency of mentoring activities reported by mentees on the PsychStart scheme.

Students reported a range of mentoring activities on the PsychStart scheme. The most popular activities were receiving careers or medical school advice, engaging in clinical shadowing, discussing psychiatry topics with mentors and being signposted to opportunities in the specialty (Fig. 3). Some students reported attending events, such as conferences, with their mentors, arranging clinical psychiatry electives, and engaging in audit or quality improvement projects and research (Fig. 3).

### Effects of mentoring and the PsychStart scheme

A majority of mentees reported improved personal (71.4%) and professional (75%) development, clinical knowledge about psychiatry (60%) and knowledge about careers in the specialty (83.6%) (Table 3).

**Table 3:** Reported effects of the PsychStart mentoring scheme

Statement	Proportion of mentees selecting each response, %				
	Strongly agree	Agree	Neutral	Disagree	Strongly disagree
‘My mentor has supported my personal development’	23.2	48.2	28.6	0	0
‘My mentor has supported my professional development’	28.6	46.4	25	0	0
‘PsychStart has increased my knowledge about careers in psychiatry’	41.8	41.8	16.4	0	0
‘PsychStart has increased my clinical knowledge about psychiatry’	29.1	30.9	34.5	5.5	0
‘PsychStart has created positive publicity for psychiatry within the medical school’	52.7	30.9	16.4	0	0

Over 80% of mentees felt that PsychStart had created positive publicity for psychiatry within the medical school, with over half of students strongly agreeing with this statement (Table 3).

### Potential links to recruitment

Approximately one-third of mentee responses (21; 32.3%) reported that PsychStart had increased their interest in psychiatry as a career. All but one of the remaining responses (43; 66.2%) reported that PsychStart had maintained their interest in a career in the specialty.

The one mentee who reported that PsychStart had decreased their interest in psychiatry as a career rated their overall scheme experience as excellent. On further clarification, this mentee had been deciding between careers in general practice and psychiatry, and through further experiences of both specialties had decided to pursue primary care. They claimed that PsychStart had helped them to make an ‘informed decision’ and that they were hoping to complete a foundation post in psychiatry.

### Qualitative feedback

In total, 57 participants provided comments on their favourite aspects of the PsychStart scheme, from which 10 key themes were identified (Table 4). Most commonly, respondents cited their individual mentoring interactions and relationships as their favourite scheme component.

**Table 4:** Major themes identified from qualitative analysis of mentees’ reported favourite aspects of the PsychStart scheme

Theme	Example quote
Positive mentoring interactions	(My mentor is) ‘easy to contact and replies thoroughly and fast to my questions about psychiatry and medical school in general’
Self-development	‘I feel the personalised aspect of having a one to one mentor is very useful for self-directed learning and attaining personal outcomes’
Feeling supported	‘Feeling it's a safe space to ask absolutely anything without being judged’
Early/enhanced clinical exposure	(I like) ‘how the scheme gives medics an early exposure to psychiatry’
Career planning	‘It has helped me feel like my decision to do psychiatry is well informed’
Extra-curricular opportunities	‘Gives me the opportunity to see areas I would not be able to see during my studies’
Scheme flexibility	‘I like the independence in choosing what I want to gain from the scheme’
Networking	‘A great way to make links with people within psychiatry’
Scheme organisation	‘The consistent follow-up from the committee to see how the relationship between mentors and mentees is going. I think that's really important’
Annual awards evening	‘I loved the awards ceremony and hearing about what everyone was doing’

Six key themes were identified from the answers of 39 respondents who offered suggestions for scheme improvement (Table 5). The most popular suggestions included greater provision of locally available mentors, more scheme publicity/advertisement and more communication prompts to mentors/mentees.

**Table 5:** Major themes identified from qualitative analysis of mentees’ suggested areas of improvement for the PsychStart scheme

Theme	Example quote
Distance from mentors	‘My mentor is quite far away. I understand that not all mentors can be close but if I didn't have a car it would be hard to meet’
Difficulty contacting mentors	‘I haven't had much contact with my mentor due to my exams/my mentor not being contactable’
More publicity	‘More advertisement of research and conference opportunities’
Regular communication prompts	‘It might be useful to have a regular email prompt to give advice or ideas for activities and prompt mentees/mentors to reflect on what they have discussed/done together’
More events	‘There could be more events that are open to everyone on the scheme to aid networking opportunities’
Structured activities	‘More structured things for mentors and mentees to do together’

### Leaving the scheme

In total, 40 mentees have left the scheme since it began, most commonly because they graduated medical school or moved location (22; 55%). Other reasons have included other commitments 3 (7.5%), wanting to explore other specialties (2; 5%) and taking a year out of medical school (1; 2.5%); 7 mentees (17.5%) left the scheme without any specific reason and 5 (12.5%) left because of poor engagement.

Of the mentors, 9 have left the scheme since its inception for various reasons: moving geographical location (3; 33.3%), other commitments (3; 33.3%), difficulty meeting their mentees owing to travel distance (2; 22.2%) and retirement (1; 11.1%).

## Discussion

The General Medical Council (GMC) mandates that all medical students must have access to educational and pastoral support and career guidance.^13^ Medical schools provide extensive educational networks for students to support academic progress, career development and well-being.

In the medical literature, the most frequently cited definition of mentoring is that provided by the Standing Committee on Postgraduate Medical and Dental Education (SCOPME), who describe it as: ‘A process whereby an experienced, highly regarded, empathetic person (the mentor) guides another (usually younger) individual (the mentee) in the development and re-examination of their own ideas, learning, and personal and professional development’.^14^ The role of a mentor is sometimes confused, and occasionally overlaps, with that of several others, including a tutor, supervisor, counsellor, advisor and role model.^15–17^ The main distinctions are the highly personal and active nature of the mentoring interaction and the focus on the individual mentee's personal and career goals, instead of professional skills.^15–18^

Over 90% of medical students perceive mentoring to be important and are keen to engage in mentoring relationships.^19,20^ Despite this, only one-third of medical students report having a mentor.^19,20^ Furthermore, there is a lack of mentoring schemes in most countries’ medical schools, including the UK.^21,22^ This lack is surprising given the evidenced widespread benefits,^15,21^ although it could be argued that there has been uneven support and guidance for potential mentors to enable them to adopt such roles.^23^

Mentors provide strong role modelling for careers and can have a significant impact on specialty choice.^21,24^ In a study including over 9000 medical students, mentors and role models were identified as the most or second-most influential factor in determining specialty selection; for ‘controllable lifestyle’ specialties, of which psychiatry was included, their influence was exceeded only by ‘lifestyle factors’.^25^

### Potential student gains from PsychStart

Our project has demonstrated that a mentoring scheme for medical students interested in psychiatry can be delivered alongside the standard curriculum and is popular among students at a large UK medical school. Participants reported several benefits from mentoring, including enhanced personal and professional development, improved careers and clinical knowledge, and feeling well supported. These benefits could apply to all students and to mentoring in other specialties, with the broader literature demonstrating similar benefits from other mentoring schemes.^21^

It is interesting that many of the qualitative comments by students highlighted generic aspects of the mentoring relationship, such as receiving personalised support, careers advice and assistance in their personal and professional development, as their favourite components of the PsychStart scheme. All students already have a personal tutor throughout the medical course, and clinical supervisors for each placement, who are also able to deliver these functions. It may be that students particularly enjoy receiving such support in the context of their desired specialty or accessing this from somebody to whom they have been closely matched on the basis of shared interests.

Many of the observed mentoring activities fulfil several selection criteria for core psychiatry training applications.^32^ For example, involvement in audit and quality improvement, completion of research, and demonstration of commitment to the specialty through arranging further clinical exposure and clinical electives with mentors. Hence, mentoring opportunities may increase employability and help students to maximise their chances of successful future training applications. This is likely to be an important attraction to the scheme as recruitment to core training becomes more competitive.

### Potential specialty gains from PsychStart

Mentoring may also facilitate increased and broadened clinical exposure to a specialty, with many students arranging shadowing opportunities with their mentors and accessing subspecialties that are less established within the standard curriculum. This is particularly useful within psychiatry, where many subspecialties, such as forensic and perinatal psychiatry, are optional or seldom taught in medical school. The Psychiatry Early Experience Programme (PEEP) has previously demonstrated the benefits of increased psychiatry exposure for young medical students, including sustained positive attitudes towards the specialty.^8^ It is unclear whether such positive attitudes extend beyond those students who engage in psychiatry enrichment programmes, but over 80% of mentees felt that PsychStart had created positive publicity for the specialty within the wider medical school, suggesting that these may permeate throughout the student body. Ajaz et al previously reported that medical students often experience ‘badmouthing’ or ‘bashing’ of psychiatry,^26^ which can deter them from entering the specialty; this highlights the importance of fostering positive attitudes and prompted the Ban the Bash campaign by the RCPsych, which aimed to identify and discourage disparaging comments about psychiatry.^27^ More recently, the College has focused on emphasising positive ‘pull factors’ that promote selection of psychiatric careers.

The most common year for students to sign up to PsychStart is year 4, which coincides with the clinical psychiatry module; hence, clinical exposure to a specialty may prompt involvement in extra-curricular opportunities. The mentoring scheme was much more popular among women students, with 75.8% of mentees being women. This is considerably larger than the approximately 55% women on the Nottingham medical course (This information was obtained by T.H. through contact with Dr Pamela Hagan at the University of Nottingham Medical School. Permission was granted from the University of Nottingham Dean of Medical Education Professor Gill Doody). It is not clear why this discrepancy exists. Prior research has associated female gender with preferential selection of psychiatry at undergraduate level;^28^ however, women have historically been less likely to receive mentorship, and to become mentors, in medical fields.^29,30^

Our scheme data provide further evidence to support the utility of mentoring in improving specialty recruitment, with over one-third of students reporting an increased interest in psychiatric careers. Our data also suggest that mentoring may help to retain, as well as promote, students’ interests in particular specialties, with just under two-thirds of students reporting a sustained interest in psychiatry on the PsychStart scheme. This is particularly important given that 22% of medical students who exhibit an early interest in psychiatry lose this interest throughout their studies.^28^ There is evidence that these benefits also exist outside of psychiatry; for example, Dorrance et al reported that a mentoring and research initiative showcasing a career as an internist in primary care settings resulted in a higher proportion of graduates opting for internal medicine training.^31^ Furthermore, mentoring has been found to increase interest in academic careers.^10,21^ Holt et al reported no significant difference in specialty choice at baseline and at their 3-year follow-up for students participating in the Psychiatry Early Experience Programme;^8^ this may suggest that, although clinical shadowing may be useful, other opportunities provided by mentorship may have a greater influence on career selection. However, further follow-up and evaluation is required to determine the long-term effects of mentoring and early clinical exposure on decisions to pursue psychiatry.

### Strengths and limitations

Our scheme data have several strengths. First, to our knowledge, this is the first published report to evaluate a formal mentoring scheme for UK medical students interested in psychiatry. Second, the high response rates to the two surveys (69.1 and 70.5%) assures us that we have gained a representative sample of students on the PsychStart programme. Third, the broad range of survey responses covering all year groups at Nottingham medical school gives strength to the generalisability of our findings.

However, we recognise that these data have the following limitations. First, they are subjective, based on students’ written reports and ratings of their mentoring relationships. The nature of the design of the evaluation did not allow for further probing about the students’ perceptions and experiences of the scheme. Furthermore, students’ stated interests may not necessarily predict future actions and result in core psychiatry training applications. Second, our data were derived from a self-selecting sample of students participating in the PsychStart scheme. We were therefore unable to draw any comparisons between students who do and do not receive mentoring in psychiatry and determine whether the scheme ‘adds value’ for those already interested in a career in the specialty. Finally, the maximum duration of mentoring relationships at the time of survey completion was just over 18 months; hence, we are unable to comment on the long-term effects of mentoring for this cohort.

### Research recommendations

We recommend that future research should examine the long-term effects of mentoring and its impact on the quantity and quality of applications to core psychiatry training. Given prior research associating mentoring with improved medical school performance and training outcomes,^21,33^ it would additionally be useful to examine the effects of mentoring in psychiatry on exam performance in the specialty. Delineating what motivates students who do and do not sign up to such mentoring schemes, and potential logistical barriers to taking part, could provide useful insights into factors both promoting and hindering engagement with psychiatry and mentoring. Further attention should be given to the experience of mentors, especially given that the concept of ‘reverse-mentoring’ is becoming increasingly recognised in the medical literature; this describes a process whereby junior medical professionals can mentor their senior colleagues, providing benefits such as enhanced understanding of digital technologies and online platforms, and improved workplace culture.^34^ Critically, it important to understand whether receiving mentoring from a psychiatrist rather than other doctors positively influences later career choice. Comparison of the impact of mentoring with other medical student enrichment activities, such as shadowing^8^ or participating in Balint groups^35^ or medical student psychotherapy schemes,^36^ needs further evaluation.

## About the authors

**Thomas Hewson**, BMBS, BMedSci, is an Academic Clinical Fellow in Psychiatry with Pennine Care NHS Foundation Trust, UK. **Nikki Thomas**, MBChB, MRCPsych, PGCME, FHEA, is a consultant psychiatrist and undergraduate clinical tutor at Cambridgeshire and Peterborough NHS Foundation Trust, and an honorary consultant psychiatrist at Nottinghamshire Healthcare NHS Foundation Trust, UK. **Kate Lovett**, BSc, MBChB, MSc, FRCPsych, Cert Clin Ed (Dist), is a consultant psychiatrist with Livewell Southwest, Plymouth, and Dean of the Royal College of Psychiatrists, London, UK. **Helen Bruce**, MBBS, BSc, MA, FRCPsych, SFHEA, is a consultant child and adolescent psychiatrist at East London NHS Foundation Trust, Professorial Teaching Fellow at the Great Ormond Street Institute of Child Health, University College London, and Associate Dean for Recruitment into Psychiatry at the Royal College of Psychiatrists, London, UK. **Derek K. Tracy**, MBBCh, BAO, MSc, FHEA, FRSA, FFFMLM, FRCPsych, is a consultant psychiatrist and clinical director with Oxleas NHS Foundation Trust, and senior lecturer at the Department of Psychosis Studies, Institute of Psychiatry, Psychology and Neuroscience, King's College London, UK.

## Supplementary material

For supplementary material accompanying this paper visit http://doi.org/10.1192/bjb.2020.107. click here to view supplementary material
