# Supplementary material for: PsychStart: a novel mentoring scheme for supporting and valuing medical students interested in psychiatry
Source: BJPsych Bull. 2021 Dec;45(6):343–50. doi: 10.1192/bjb.2020.107 (PMC8727381; doi:10.1192/bjb.2020.107)
Supplement: Supplementary file 1 [file S2056469420001072sup001.zip › S2056469420001072sup001.docx]

**Supplementary Material**

**Appendix 1 – Questions Included in PsychStart Evaluation Surveys**

1. Please provide your name and year group below:

[free text]

1. For what duration have you been a member of the PsychStart Scheme?

<3 months; 3-6 months; 6-9 months; 9-12 months; 12-18 months; >18 months

1. Overall how would you rate your experience on PsychStart?

Excellent; Good; Average; Poor; Very Poor

1. Overall, how would you rate your individual mentoring relationship with your mentor?

Excellent; Good; Average; Poor; Very Poor

1. On average, how often are you in contact with your mentor?

Weekly or more often; Monthly; Every 3 months (once a semester); Every 6 months; Yearly

1. How many times have you met your mentor face-to-face?

[free text]

1. What activities have you been able to engage in with your mentor? (Please select all that apply)

Careers advice; Medical school advice; Clinical shadowing; Audit participation; Research participation; Elective organisation; Signposting to opportunities; Discussion about psychiatry topics; Attending events together (e.g. conferences); Other (please state) [free text].

1. "I believe I was well matched to my mentor"

Strongly agree; Agree; Neutral; Disagree; Strongly Disagree

1. “My mentor has helped to support my personal development"

Strongly agree; Agree; Neutral; Disagree; Strongly Disagree

1. "My mentor has helped to support my professional development"

Strongly agree; Agree; Neutral; Disagree; Strongly Disagree

1. How would you rate the organisation of PsychStart?

Excellent; Good; Average; Poor; Very Poor

1. How would you rate the level of support available from the scheme organisers on PsychStart?

Excellent; Good; Average; Poor; Very Poor

1. "PsychStart has increased my knowledge about psychiatry as a career"

Strongly agree; Agree; Neutral; Disagree; Strongly Disagree

1. 'PsychStart has increased my clinical knowledge about psychiatry"

Strongly agree; Agree; Neutral; Disagree; Strongly Disagree

1. "PsychStart has created positive publicity for psychiatry within the medical school"

Strongly agree; Agree; Neutral; Disagree; Strongly Disagree

1. Please comment below your favourite aspects/components of PsychStart.

[free text]

1. Please comment below areas of improvement for PsychStart

[free text]

1. How has PsychStart changed your level of interest in psychiatry as a career?

Increased my interest; Maintained my interest; Decreased my interest

1. Do you wish to remain on the scheme?

Yes; No

1. Do you consent to the use of your anonymised data as part of future PsychStart promotional material and research?

Yes; No

1. Any other comments?

[free text]
